# Naturally-Acquired Immune Response against *Plasmodium vivax* Rhoptry-Associated Membrane Antigen

**DOI:** 10.1371/journal.pone.0148723

**Published:** 2016-02-17

**Authors:** Siriruk Changrob, Bo Wang, Jin-Hee Han, Seong-Kyun Lee, Myat Htut Nyunt, Chae Seung Lim, Takafumi Tsuboi, Patchanee Chootong, Eun-Taek Han

**Affiliations:** 1 Department of Clinical Microbiology and Applied Technology, Faculty of Medical Technology, Mahidol University, Bangkok, Thailand; 2 Department of Medical Environmental Biology and Tropical Medicine, School of Medicine, Kangwon National University, Chuncheon, Gangwon-do, Republic of Korea; 3 Department of Clinical Laboratory, The First Affiliated Hospital of Anhui Medical University, Hefei, Anhui, People’s Republic of China; 4 Department of Medical Research, Yangon, Myanmar; 5 Department of Laboratory Medicine, College of Medicine, Korea University Guro Hospital, Guro Gu, Seoul, Republic of Korea; 6 Division of Malaria Research Proteo-Science Center, Ehime University, Matsuyama, Ehime, Japan; Universidade Federal de Minas Gerais, BRAZIL

## Abstract

Rhoptry-associated membrane antigen (RAMA) is an abundant glycophosphatidylinositol (GPI)-anchored protein that is embedded within the lipid bilayer and is implicated in parasite invasion. Antibody responses against rhoptry proteins are produced by individuals living in a malaria-endemic area, suggesting the immunogenicity of *Plasmodium vivax* RAMA (PvRAMA) for induction of immune responses during *P*. *vivax* infection. To determine whether PvRAMA contributes to the acquisition of immunity to malaria and could be a rational candidate for a vaccine, the presence of memory T cells and the stability of the antibody response against PvRAMA were evaluated in *P*. *vivax*-exposed individuals. The immunogenicity of PvRAMA for the induction of T cell responses was evaluated by *in vitro* stimulation of peripheral blood mononuclear cells (PBMCs). High levels of interferon (IFN)-γ and interleukin (IL)-10 cytokines were detected in the culture supernatant of PBMCs, and the CD4^+^ T cells predominantly produced IL-10 cytokine. The levels of total anti-PvRAMA immunoglobulin G (IgG) antibody were significantly elevated, and these antibodies persisted over the 12 months of the study. Interestingly, IgG1, IgG2 and IgG3 were the major antibody subtypes in the response to PvRAMA. The frequency of IgG3 in specific to PvRAMA antigen maintained over 12 months. These data could explain the immunogenicity of PvRAMA antigen in induction of both cell-mediated and antibody-mediated immunity in natural *P*. *vivax* infection, in which IFN-γ helps antibody class switching toward the IgG1, IgG2 and IgG3 isotypes and IL-10 supports PvRAMA-specific antibody production.

## Introduction

One of the major global public health problems is malaria, a life-threatening blood disease caused by the *Plasmodium* parasite, which is transmitted to humans by female *Anopheles* mosquitoes. Among the five *Plasmodium* species known to infect humans, *Plasmodium vivax* has a wide geographical distribution in Southeast and East Asia, which causes approximately 66% of the total global vivax malaria burden [1, 2]. Although *P*. *vivax* is less virulent than *P*. *falciparum*, it presents as a characteristic unique relapsing disease that may reactivate after months or years without symptoms. Because the number of reports of *P*. *vivax* resistance to first-line antimalarial drug treatment has significantly increased, research into the development of a vaccine is considered be an important approach to blocking malaria transmission [3].

Evidence from both immunization experiments in animals and from human studies has suggested the possibility of vaccination against malaria [4–8]. Effective targets for inducing a protective immune response have been identified from each of the three stages of the life cycle of *Plasmodium*: the pre-erythrocytic stage, the asexual erythrocytic stage, and the sexual stage. Vivax vaccine candidates have been developed based on orthologues of *P*. *falciparum* antigens, because of the limitations of effective and continuous *in vitro* culture of *P*. *vivax*. Candidate vaccines against pre-erythrocytic stage antigen *P*. *vivax* circumsporozoite surface protein (PvCSP) and sexual stage antigen *P*. *vivax* ookinete surface protein 25 (Pvs25) have been evaluated in Phase I clinical trials [9], while blood stage vaccine candidates such as merozoite surface protein 1–42 (MSP1_42_), merozoite surface protein 1–19 (MSP1_19_), the N-terminal fragment of merozoite surface protein 1 (Pv200L), Duffy-binding protein region II (DBPII), and apical membrane antigen 1 (AMA-1) are undergoing preclinical studies [10, 11].

Besides the role of the merozoite surface proteins, the secretory organelles (micronemes, rhoptries, and dense granules) of the apical complex are involved in the invasion of erythrocytes [12]. The rhoptries are significant structures that have been implicated in the parasitophorous vacuole and in formation of the parasitophorous vacuole membrane [12, 13]. Antibody responses against the rhoptry proteins, rhoptry-associated protein (RAP)-1, RAP-2, RAP-3, and the high-molecular-weight complex of rhoptry protein-3 (RhopH3) were detected in a population of individuals living in malaria-endemic areas [14–17]. Antibodies against RAP-1 and RAP-2 antigen inhibited *P*. *falciparum* growth in an *in vitro* erythrocyte invasion assay [18]. Importantly, RAP-1 protein was reported to be protective against a lethal blood-stage infection of *P*. *falciparum* malaria response in an immunized monkey [19]. The immunogenicity of rhoptry-associated membrane antigen (RAMA) was identified and characterized in individuals exposed to *P*. *falciparum* [13]. The immunodominant p60 form of the RAMA epitope (RAMA-E) showed the highest prevalence in the antibody response. This epitope strongly boosted a humoral response that persisted for up to 28 days postinfection. Interestingly, high levels of anti-RAMA-E IgG3-type antibodies were detected in protected individuals who had no detectable parasites even though they lived in a high-incidence malaria area, indicating that this antibody response might be associated with protection against *P*. *falciparum*. Recently, the well-characterized *P*. *falciparum* RAMA (PfRAMA) orthologue was used to identify PvRAMA [20, 21]. A high rate of positivity for anti-PvRAMA antibodies was reported in *P*. *vivax*-infected patients, implying that this antigen could be a serological marker of recent exposure to vivax malaria. To evaluate PvRAMA antigen as a vaccine candidate, its ability to elicit an immune response, including the development of memory T cells and specific antibody against PvRAMA, and the stability of the anti-PvRAMA antibody response were assessed in *P*. *vivax*-exposed individuals from malaria-endemic areas.

## Results

### Cytokine profile in culture supernatant of peripheral blood mononuclear cells (PBMCs)

**Fig 1:**
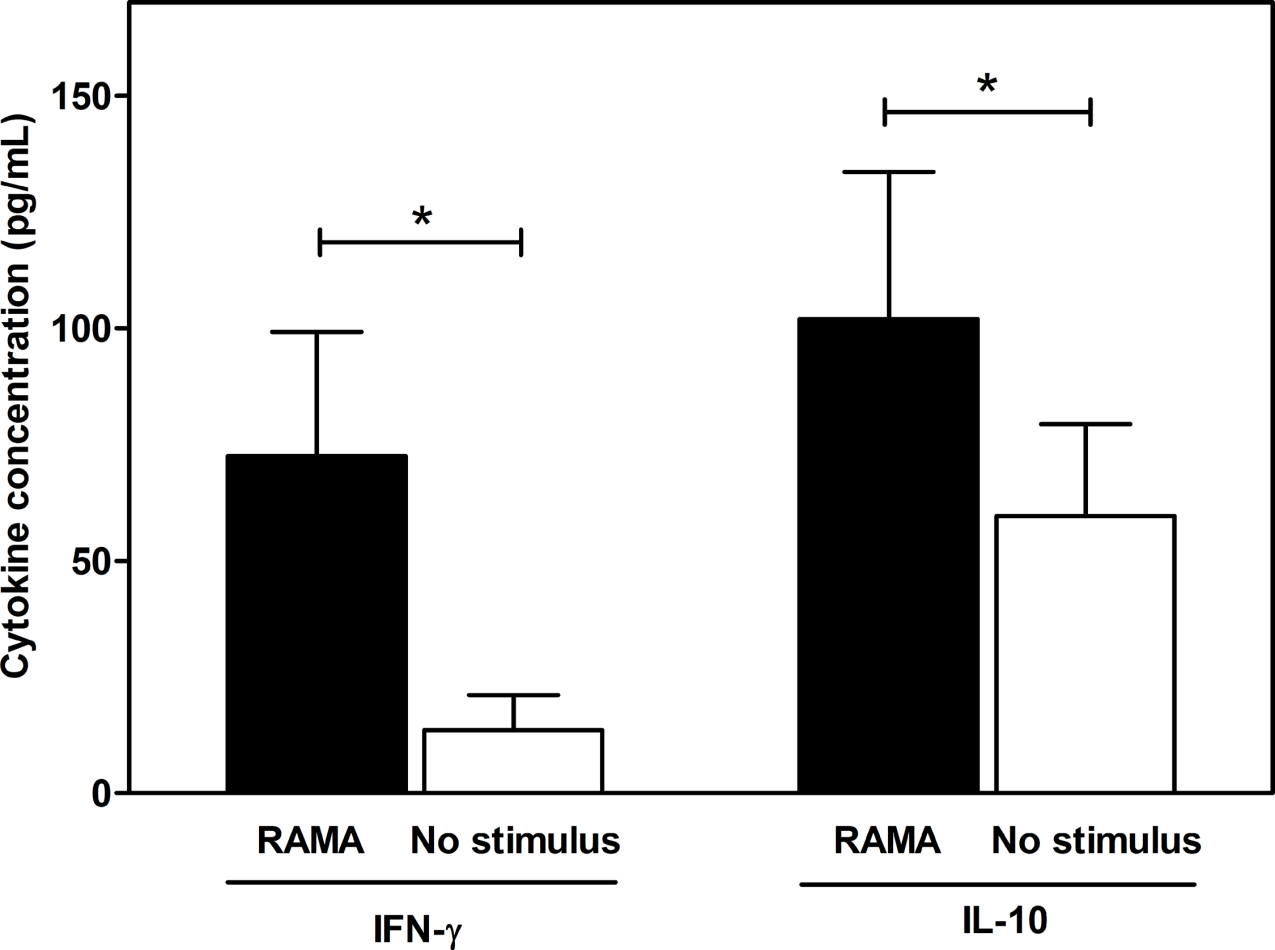
Profile of IFN-γ and IL-10 in 96-h culture supernatants. PBMCs from individuals who had recovered from *P*. *vivax* infection 8–10 weeks previously were stimulated with PvRAMA antigen, media alone (negative control), or PHA (positive control) for 96 h. The concentrations of cytokines in supernatants from stimulated cells were measured by enzyme-linked immunosorbent assay (ELISA). The histograms represent the mean cytokine levels from PBMCs of ten *P*. *vivax*-recovered subjects. The significance of differences was assessed using the Wilcoxon matched-pairs signed-rank test.

The presence of effector T lymphocytes whether they are Th1 or Th2 cell responses to PvRAMA was assessed by measurement of IL-10 and IFN-γ cytokines in the culture supernatant following antigen stimulation of PBMCs. The IFN-γ concentrations produced by PvRAMA-stimulated PBMCs from patients who had recovered from *P*. *vivax* infection were significantly higher than those produced by unstimulated control cells (PvRAMA = 72.45 ± 80.07 pg/ml, (mean ± standard deviation [SD]), unstimulated = 13.59 ± 22.53 pg/ml, *P* = 0.0078, Fig 1). For IL-10 cytokine, the levels of PvRAMA-induced IL-10 were also significantly higher than those in unstimulated controls (PvRAMA = 101.90 ± 105.14 pg/ml, unstimulated = 59.64 ± 65.33 pg/ml, *P* = 0.0019, Fig 1). PBMCs from acutely infected *P*. *vivax* patients showed no significant IFN-γ and IL-10 responses to PvRAMA antigen compare to unstimulated control cells (data not shown). Production of both IFN-γ and IL-10 production in response to phytohemagglutinin (PHA) was threefold higher than that in response to PvRAMA antigen.

### Effector response of PvRAMA-specific CD4^+^ and CD8^+^ T cells

**Fig 2:**
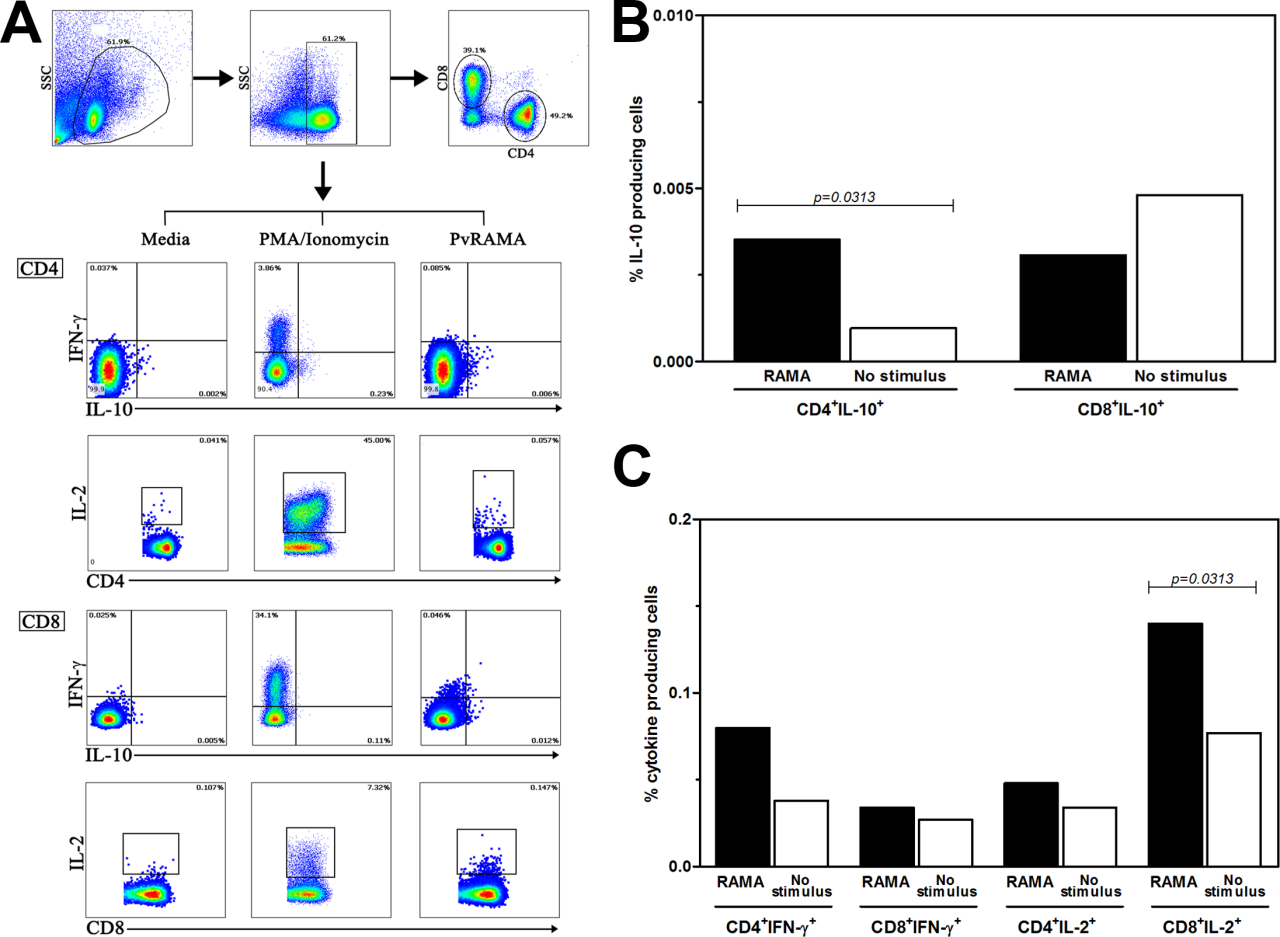
CD4^+^ and CD8^+^ T cell response specific for PvRAMA antigen assessed by intracellular cytokine staining and multiparameter flow cytometry. (A). Gating strategy for analysis by multiparameter flow cytometry to identify the T-cell response of PBMCs from *P*. *vivax*-recovered subjects to PvRAMA or media alone (negative control) or to phorbol myristate acetate (PMA)/ionomycin (positive control). The bar graphs represent the results obtained for PvRAMA-specific (B) IL-10-producing cells, (C) IFN-γ-producing cells and IL-2-producing cells. The significance of differences in the percentages of cytokine-producing cells were determined with the Wilcoxon matched-pairs signed-rank test.

To identify cell phenotypes from effector responses to PvRAMA antigen in *P*. *vivax* infection, the levels of IFN-γ- and IL-10-producing cells induced in response to PvRAMA stimulation were analyzed using flow cytometry (Fig 2A). After stimulation, CD4^+^ T cells were major cell population produced IL-10 cytokine against PvRAMA antigen (PvRAMA = 0.004 ± 0.007%, (median ± standard deviation [SD]), unstimulated = 0.001 ± 0.002%, *P* = 0.0313, Fig 2B). Both CD4^+^ and CD8^+^ T cells were not IFN-γ-producing cells in response to PvRAMA antigen (CD4^+^; PvRAMA = 0.080 ± 0.036%, unstimulated = 0.038 ± 0.022%, *P* = 0.0625, CD8^+^; PvRAMA = 0.034 ± 0.049%, unstimulated = 0.027 ± 0.014%, *P* = 0.5000, Fig 2C). The detection of IL-2-producing cells showed that both CD4^+^ and CD8^+^ T cells produced IL-2 cytokine after PvRAMA stimulation and CD8^+^ effector T cells were the main IL-2 source (CD4^+^; PvRAMA = 0.048 ± 0.036%, unstimulated = 0.034 ± 0.023%, *P* = 0.1563, CD8^+^; PvRAMA = 0.140 ± 0.080%, unstimulated = 0.077 ± 0.078%, *P* = 0.0313, Fig 2C).

### Serological response to PvRAMA

**Fig 3:**
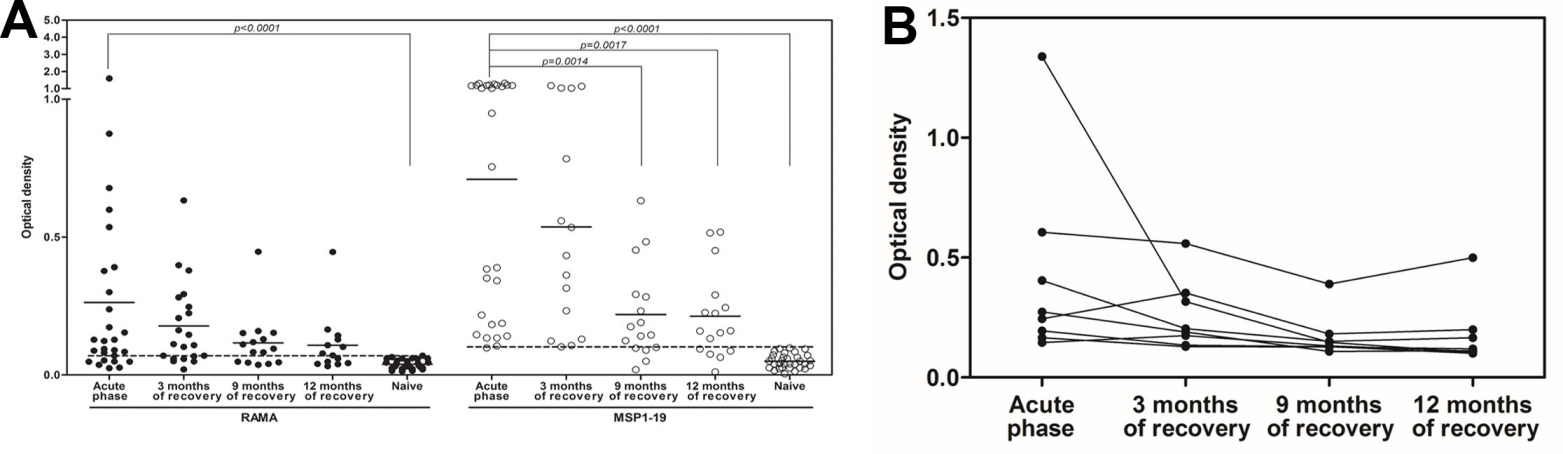
Levels of naturally acquired antibody against PvRAMA in *P*. *vivax*-exposed individuals. (A). The level of PvRAMA and PvMSP1-19-specific total IgG in 77 serum samples from *P*. *vivax*-exposed individuals, including 27 samples from the acute phase, 21 samples collected 3 months after recovery, 15 samples collected 9 months after recovery, and 14 samples collected 12 months after recovery, plus 32 serum samples from malaria-naïve individuals. (B). Stability of the antibody response against PvRAMA in a group of recovered patients at different time points (acute phase, 3 months, 9 months and 12 months of recovery). The cutoff value for seropositivity was an OD greater than the mean plus two SD of that for the naïve controls (PvRAMA cutoff OD = 0.071, PvMSP1-19 cutoff OD = 0.101). Antibody levels in different groups were compared using the Mann–Whitney *U* test.

To measure the antibody response to PvRAMA during *P*. *vivax* infection, the levels of total IgG specific for purified recombinant PvRAMA and PvMSP1-19 were analyzed in plasma samples from acutely *P*. *vivax*-infected patients. Antibody specific to PvRAMA in acute *P*. *vivax* patients were significantly higher than those in naïve controls (*P*. *vivax* patient, optical density [OD] = 0.263 ± 0.348, (mean ± standard deviation [SD]), Naïve controls, OD = 0.040 ± 0.018, *P* < 0.0001, Fig 3A) and PvMSP1-19 (*P*. *vivax* patient, OD = 0.709 ± 0.485, Naïve control, OD = 0.050 ± 0.026, *P* < 0.0001, Fig 3A). The result confirms the ability of PvRAMA and PvMSP1-19 to induce antibody responses during natural *P*. *vivax* exposure. To investigate the stability of these anti-RAMA antibody responses further, the antibody levels specific to PvRAMA and PvMSP1-19 antigens were measured in individuals followed up at 3, 9, and 12 months after they recovered from *P*. *vivax* infection. The results show that antibody levels in response to PvRAMA antigen presented in *P*. *vivax* recovery at least 12 months (Fig 3A). The seropositivities in a period of 3, 9, and 12 months after treatment were observed 61.9%, 73.3% and 50%, respectively. In contrast, anti-PvMSP1-19 antibody levels in 9 month *P*. *vivax* recovery were significantly lower than acute *P*. *vivax* patients (Fig 3A) and 100%, 68.8% and 68.8% of individuals showed seropositivity in a period of 3, 9 and 12 months following treatment, respectively (Table 1). Additionally, the following anti-PvRAMA antibody levels in eight individuals of acute and in a period of 3, 9, and 12 month following treatment showed that antibody responses to this antigen still presented in individuals. All recovery subjects who were seropositive at the acute phase remained seropositive to PvRAMA over the 12 months of the study (Fig 3B). No correlation between anti-PvRAMA antibody levels with gender and age were observed in this study (data not shown).

**Table 1:** Prevalence and magnitude of anti-PvRAMA and anti-PvMSP1-19 antibody responses.

Time point^a^	PvRAMA						PvMSP1-19					
	Percent positive^b^ (%)	OD values					Percent positive^b^ (%)	OD values				
		Min^c^	Max^d^	Mean^e^	SD^f^	*P*-value^g^		Min^c^	Max^d^	Mean^e^	SD^f^	*P*-value^g^
Acute phase	74.1	0.025	1.595	0.263	0.348	< 0.0001	96.4	0.098	1.310	0.709	0.485	< 0.0001
3 months of recovery	61.9	0.020	0.633	0.178	0.153	NS	100.0	0.102	1.163	0.536	0.396	NS
9 months of recovery	73.3	0.037	0.447	0.116	0.101	NS	68.8	0.019	0.632	0.219	0.172	0.0014
12 months of recovery	50.0	0.032	0.446	0.107	0.106	NS	68.8	0.011	0.518	0.212	0.158	0.0017

^a^ Time point: the time at which blood samples were collected after recovery from *P*. *vivax* infection.

^b^ Percent positive: the percentage of seropositive individuals who had OD values greater than the cutoff value (mean ± 2 SD of the OD value of malaria-naïve individuals).

^c^ Min: the lowest antibody level at each time point.

^d^ Max: the highest antibody level at each time point.

^e^ Mean: the average of antibody level presented as mean OD value at each time point.

^f^ SD: the standard deviation of antibody levels at each time point.

^g^ *P* value of the difference between the mean antibody levels of *P*. *vivax*-infected subjects with recovery phase or malaria-naïve compared using the Mann–Whitney *U* test. NS: not significant.

### Anti-PvRAMA IgG antibody isotypes

**Fig 4:**
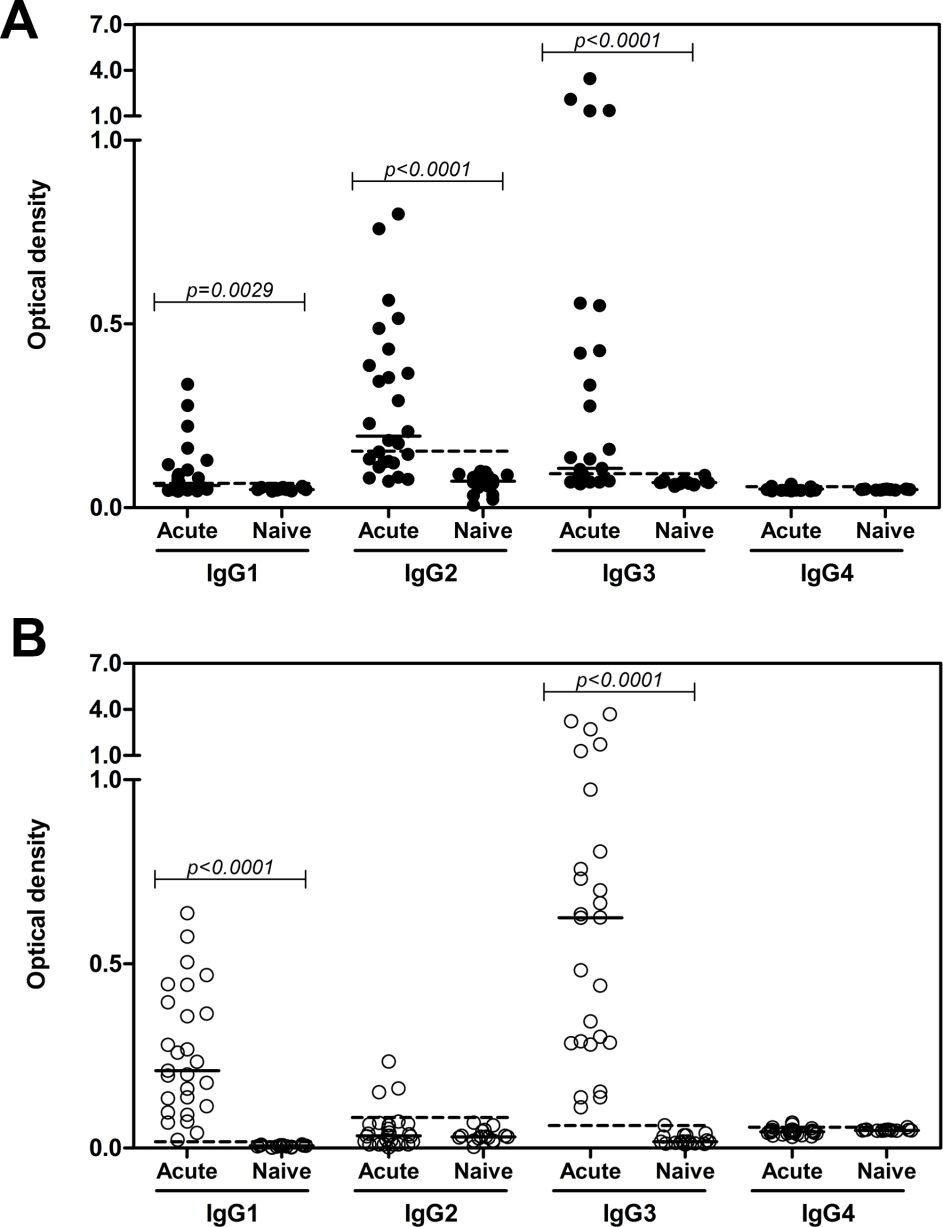
IgG isotype antibody response to PvRAMA. (A). The levels of PvRAMA- and (B) PvMSP1-19-specific antibodies of each IgG isotypes were determined in plasma from acutely *P*. *vivax*-infected patients and naïve individuals by ELISA. Vivax patients (*n* = 27) and naïve controls (*n* = 16) were randomly selected for IgG isotype prevalence studies. The cutoff value for each IgG isotype was calculated from the mean plus 2SD of the OD at 450 nm of malaria-naïve control as shown by dashed line (PvRAMA; IgG1 = 0.058, IgG2 = 0.119, IgG3 = 0.082, IgG4 = 0.051, PvMSP1-19; IgG1 = 0.011, IgG2 = 0.066, IgG3 = 0.050, IgG4 = 0.053). The significance of differences between the levels of IgG isotypes were assessed using the Mann–Whitney *U* test.

To evaluate whether specific IgG subclasses were sustained in antibody response to PvRAMA antigen, the prevalence of PvRAMA and PvMSP1-19-specific antibodies of different IgG isotypes were evaluated in acutely *P*. *vivax*-infected patients and in a period of 3, 9, 12 months following treatment. In plasma collected from acute *P*. *vivax* infection, the percentages of sera positive against PvRAMA for IgG1, IgG2, IgG3, and IgG4 isotype antibodies were 55.6%, 77.8%, 63%, and 51.9%, respectively. The anti-RAMA antibodies in *P*. *vivax* patients were predominantly of the IgG2 and IgG3 isotype, followed by IgG1, whereas IgG4 was barely measurable (Table 2 and Fig 4A). The levels of IgG1, IgG2, and IgG3 isotype antibodies against PvRAMA were significantly higher in *P*. *vivax*-infected patients than in naïve controls. Moreover, analysis of the IgG3 isotype response in acute patients showed that the level of this antibody isotype in each individual was significantly higher than that of IgG1, and that there was a significant correlation between the levels of these two cytophilic isotypes (*r*_*s*_ = 0.7597, *P* < 0.0001, data not shown). Similarly, for the noncytophilic isotypes, an increased IgG2 level was significantly associated and correlated with low IgG4 levels (*r*_*s*_ = –0.4817, *P* < 0.0001, data not shown). In PvMSP1-19 antigen, 100%, 22.2%, 100% and 14.8% of individuals were seropositive at acute *P*. *vivax* infection for IgG1, IgG2, IgG3, and IgG4 isotype antibodies, respectively. IgG1 and IgG3 were the predominant subclasses that recognized PvMSP1-19 protein (Fig 4B).

**Table 2:** Comparison of anti-PvRAMA antibody isotypes in *P*. *vivax*-exposed individuals and malaria-naïve individuals.

Time point^a^	IgG1		IgG2		IgG3		IgG4	
	Percent positive^a^ (%)	Median^b^	Percent positive^a^ (%)	Median^b^	Percent positive^a^ (%)	Median^b^	Percent positive^a^ (%)	Median^b^
Acute phase	55.6	0.060	77.8	0.195	63.0	0.107	51.9	0.051
3 months of recovery	50.0	0.052	53.3	0.122	73.3	0.114	20.0	0.048
9 months of recovery	42.9	0.043	46.7	0.070	60.0	0.102	46.7	0.048
12 months of recovery	35.7	0.043	28.6	0.067	42.9	0.077	28.6	0.048
Naïve	0.0	0.050	0.0	0.075	0.0	0.068	0.0	0.049

^a^ Percent positive: the percentage of seropositive individuals who had an OD value greater than the cutoff value.

^b^ Median: the median antibody level presented as median OD value.

**Fig 5:**
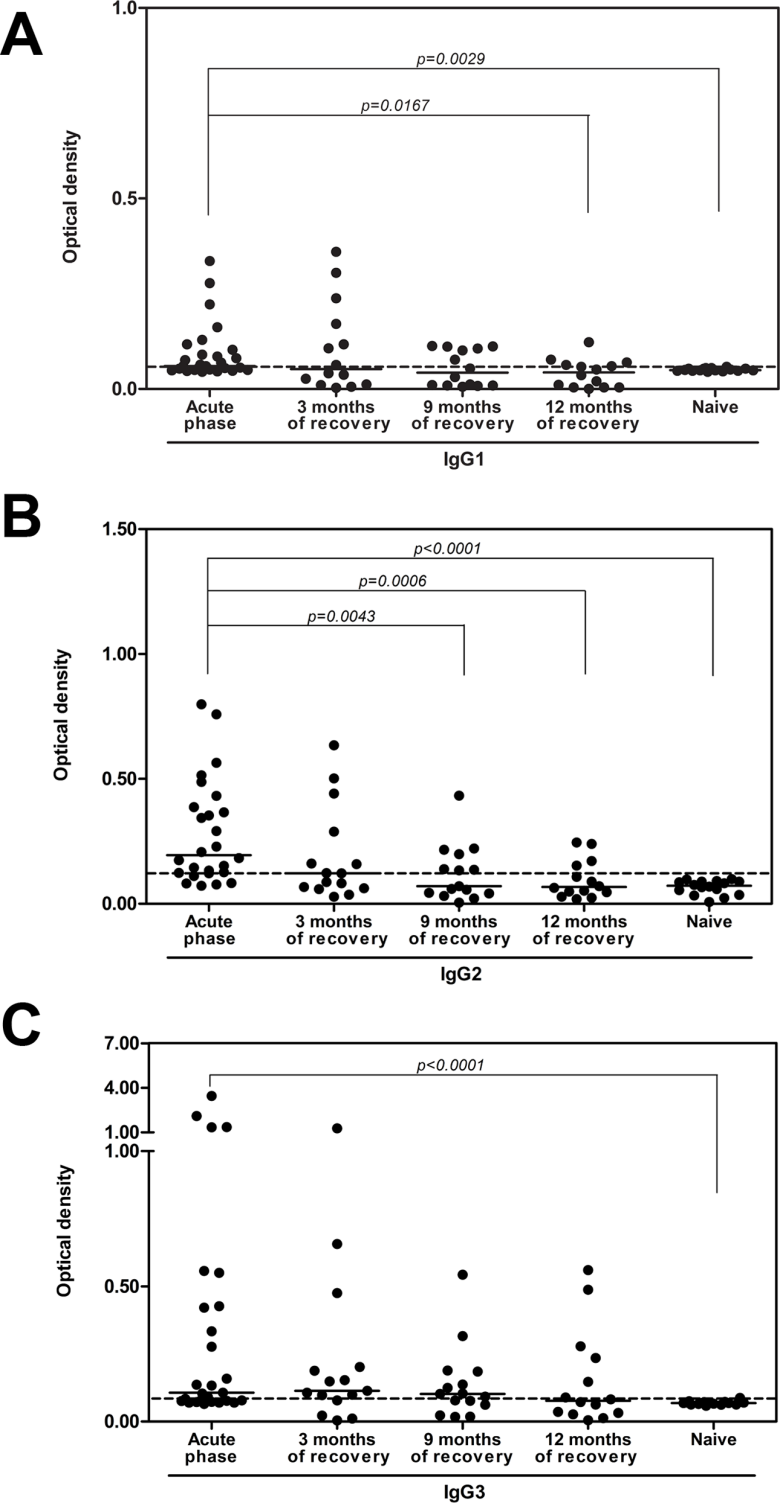
Stability of IgG1, IgG2 and IgG3 subclasses specific to PvRAMA antigen. The levels of IgG subclasses, (A) IgG1, (B) IgG2, (C) IgG3 in plasma of the individuals during acute infection and after 3, 9, 12 months of recovery, respectively. The cutoff value for seropositivity was an OD greater than the mean plus 2SD of that from naïve controls as shown by dashed line (IgG1 = 0.058, IgG2 = 0.119, IgG3 = 0.082, IgG4 = 0.051). IgG subclass levels in different groups were compared using the Mann-Whitney *U* test.

Because IgG1, IgG2 and IgG3 showed significantly higher in acute patients, we then measured stability of these IgG subclasses specific to PvRAMA antigen in individuals of acute *P*. *vivax* patients in period of 3, 9 and 12 months following treatment. The result found that 42.9%, 28.6% and 50.0% of individuals maintained positive of IgG1, IgG2 and IgG3 at 12 months (Fig 5A–5C). The frequency of individual with specific IgG1 and IgG2 responses was still significantly higher at 9 months and at 3 months, respectively compared to these antibody levels in acute patients (Fig 5A and 5B). Interestingly, the prevalence of IgG3 specific to PvRAMA antigen did not change at 12 months (Fig 5C).

## Discussion

Rhoptry proteins of *Plasmodium* parasites have been implicated in the invasion of erythrocytes. There is growing evidence that rhoptry proteins are an important target for a protective humoral immune response against malaria. In *P*. *vivax*, RAMA was first identified by screening expression libraries with patient sera, which showed a high prevalence of specific antibodies in >90% of sera from *P*. *vivax* patients [20]. The C-terminal region of PvRAMA is antigenic, and antibodies against this region are associated with immunity during infection. These data suggested that PvRAMA could be a serological marker of recent exposure to *P*. *vivax* infection. Here, we investigated cellular and antibody mediated immune responses to recombinant PvRAMA protein in *P*. *vivax*-exposed patients. A significantly higher level of production of IFN-γ and IL-10 induced by PvRAMA antigen stimulation of PBMCs was observed in PBMC cultures from *P*. *vivax*-recovered subjects. CD4^+^ T cells were the major source of IL-10, whereas the high levels of IFN-γ were not produced by effector CD4^+^ and CD8^+^ memory cells. Analysis of the antibody responses against PvRAMA showed that there was a persistence of total IgG anti-PvRAMA antibody levels in *P*. *vivax* infection as the antibody was detected in acutely infected patients and at 3, 9 and 12 months after recovery from *P*. *vivax*, respectively. IgG1, IgG2 and IgG3 were the predominant antibody isotypes generated in response to PvRAMA. Moreover, a study of IgG isotype response in individuals of acute patients showed a strong correlation between the levels of anti-PvRAMA IgG2 and IgG4 isotypes, as well as between the levels of IgG3 and IgG1, which showed that IgG2 and IgG3 increased when the levels of IgG4 and IgG1 were low (data not shown). This observation suggested that the PvRAMA antigen has some characteristics that can trigger effector memory T cells and influence the patterns of IgG isotype switching to IgG1, IgG2 and IgG3 during *P*. *vivax* infection.

The level and the IgG isotype profile of antibody against blood stage antigens are associated with the quality of protective immunity to *P*. *vivax* malaria, which in turn can result in a reduction in parasitemia and clinical malaria symptoms [22]. Antibodies to most asexual blood stage antigens have been reported to be easily induced by *P*. *vivax* infection but are apparently short-lived in the absence of repeat infection [23]. However, a previous study demonstrated the presence of high-titer antibody responses to key vaccine-candidate blood stage antigens including PvAMA-1, PvMSP1_19_, and PvDBP [24]. Similarly, antibodies to anti-*P*. *vivax* tryptophan-rich antigen persisted stably for 5 to 12 years in Chinese residents who lived in low malaria-endemic areas [25]. Recently, *P*. *vivax* patients living in an endemic area of the Republic of Korea showed significant rates of seropositivity for antibody against PvRhopH2 (>59%) and PvRAMA (>90%) [20, 21]. In the present study, we demonstrated a high prevalence of anti-PvRAMA antibodies in Thai residents, which persisted for over one year although the antibody level gradually decreased. Some patients, aged 30–68 years, displayed a consistent level of antibodies over all periods of convalescence. This may be a feature of individuals who have lived in a malaria-endemic area for a long time, leading to the long-lived persistence of anti-RAMA antibodies. However, further studies of the correlation between the levels of antibody and the frequency of circulating memory B cells specific for PvRAMA antigen are required to elucidate the factors leading to the induction of a long-lived antibody response.

It is well established that T-cells play a crucial role in driving the pattern of antibody isotype switching. Different malaria antigens are capable of inducing different IgG isotype profiles [26]. The protective activity of the cytophilic isotypes, IgG1 and IgG3, is believed to eliminate parasites by means of opsonization of the infected red blood cells and cooperation between cells by the process called antibody-dependent cellular inhibition [27]. Malaria blood-stage antigens, such as MSP5, MSP1_19_, and DBPII, in *P*. *vivax* mostly tend to generate antibody responses that are polarized towards either IgG1 or IgG3 isotypes [28–32]. In particular, IgG3 has the highest binding affinity for the Fcγ receptor on the surface of monocytes, which strongly induces phagocytosis and complement fixing [33]. The prevalence of IgG3 is also influenced by the maturity of the immune system (age) or repeated exposure to antigen [26]. PvMSP1_19_ and *P*. *vivax* reticulocyte binding protein 1_1392–2076_ predominantly induced noncytophilic isotypes IgG2 and IgG4 in patients from a malaria-endemic area of Brazil [34, 35]. Here, we demonstrated that IgG1, IgG2 and IgG3 were the predominant IgG isotypes present in the response against PvRAMA antigen whereas IgG1 and IgG3 showed high immune responses to PvMSP1-19 antigen. The higher levels of PvRAMA-induced IgG2 and IgG3 appear to be correlated with lower levels of IgG1 and IgG4 during acute *P*. *vivax* infection of individuals 18–64 years old. Interestingly, seropositivity of PvRAMA-specific IgG3 maintained at least 12 months after treatment. These data indicated that antibody responses to PvRAMA antigen were associated with immunoglobulin IgG isotype switching during natural *P*. *vivax* exposure. A study of association between IgG isotype responses and protective activity against malaria was shown in previous studies of *P*. *falciparum* ring-infected erythrocyte surface antigen and *P*. *falciparum* MSP2 antigen showed that IgG2 antibodies against these antigens were involved in the protection against malaria infection and that their levels were associated with age [36]. Nevertheless, in this study, we could not confirm whether the increase in levels of IgG2 and IgG3 antibodies against PvRAMA was completely associated with the degree of protective immunity to *P*. *vivax*. Further studies are required to determine the correlation of anti-PvRAMA IgG2 and IgG3 antibody levels with a reduction in parasitemia and the rate of reinfection with *P*. *vivax*.

In addition to antibody-mediated immunity, cell-mediated immunity also plays a crucial role in antimalarial protection [22, 37]. The function of blood stage antigen-specific CD4^+^ T cells in *P*. *vivax* patient during acute infection and after recovery from the infection has been studied widely [38–42]. In this study, we found that PvRAMA stimulation triggered a host immune response and elevated levels of production of IFN-γ and IL-10 by PBMC collected from individuals 8–10 weeks following treatment. These results suggest that individuals naturally infected by *P*. *vivax* can generate effector memory cells specific to PvRAMA antigen. The re-stimulation with PvRAMA triggers effector memory cell response by IFN-γ and IL-10 production. Because high levels of IFN-γ support IgG isotype switching, a high IFN-γ levels after antigen stimulation could suggest that the IFN-γ-producing cell response induced protective IgG isotype switching toward IgG1, IgG2 and IgG3 during *P*. *vivax* infection. However, the IFN-γ production responded to PvRAMA stimulation was not produced by CD4^+^ T cells. It may secreted from other sources of human memory cells such as CD8^+^ T cells and γδ T cells besides CD4^+^ T cells [43, 44]. Additionally, here we also demonstrated a significant elevation of IL-10 levels in PBMC culture and of the percentage of IL-10-producing CD4^+^ T cells after PvRAMA stimulation. Increased IL-10 production upon PvRAMA stimulation may help the induction of memory B cells or long-lived plasma cells because we showed that anti-PvRAMA antibodies can persist for at least 12 months. Consistent with this hypothesis, Wipasa *et al*. showed that long-term maintenance of antibody and of memory B cells were linked, because after *P*. *falciparum* exposure of individuals who had specific memory B cells, IL-10 responses were detected that were maintained for at least 6 years. This led to the suggestion that the half-life of those IL-10 responses was indefinite [24, 39].

In conclusion, PvRAMA has the ability to induce a potent effector T cell response and a long-lived antibody response following *P*. *vivax* infection. High levels of IFN-γ induced by PvRAMA stimulation and the predominance of IgG1, IgG2 and IgG3 specific for PvRAMA during acute infection could imply that effector IFN-γ-producing cells play an important role in immunoglobulin IgG isotype switching in response to this antigen. A substantial antibody response was detected for over 12 months and CD4^+^ IL-10-producing cells were observed during convalescence, suggesting that Th2 cells were involved in long-term memory B cell responses to PvRAMA antigen. All data suggest that PvRAMA could be an effective vaccine candidate because it has the ability to induce both potent effector memory T cells and a long-lived antibody response in *P*. *vivax*-exposed individuals living in a malaria-endemic area.

## Materials and Methods

### Ethics statement

Ethics approval was obtained from the Committee on Human Rights Related to Human Experimentation, Mahidol University, and the Ministry of Health, Thailand (MUIR 2012/079.2408). The patients signed informed consent to enrolment in this study before blood samples were collected. Human immunodeficiency virus-infected individuals, pregnant women, and children <18 years old were excluded from this study.

### Study population and blood collection

To evaluate the cellular immune response to PvRAMA, PBMCs were isolated from 10 *P*. *vivax*-experienced individuals who had recovered from their last *P*. *vivax* malaria episode about 8 to 10 weeks prior to sample collection. The subjects were 18–63 years old (38 ± 14 years old) and comprised 3 women and 7 men. To assess the prevalence and longevity of anti-PvRAMA antibody, blood samples were collected from 27 acutely *P*. *vivax*-infected patients and at 3, 9, and 12 months after their recovery, and plasma was isolated (Table 3). All study subjects were from Rap Ro, a village near the Myanmar border, which is in a malaria-endemic area of southern Thailand. Blood samples for malaria-naïve controls were obtained from 32 healthy volunteers who live in Bangkok and had no history of exposure to *Plasmodium* parasites. Venous blood samples were collected in Vacuette Heparin tubes (Greiner Bio-One, Monroe, NC, USA) and transported to the laboratory within 6 h. Thick and thin blood smears were used to examine for the presence of vivax parasites in acute infected *P*. *vivax* patients and in recovery subject at 3, 9, 12 months following treatment.

The selecting criteria of acute *P*. *vivax* infected volunteers were as followings: (1) systolic blood pressure was not less than 90 mm, (2) body temperature was not higher than 40°C, (3) Hematocrit was not less than 25% (4) The patients did not receive treatment with corticoids or non-steroidal anti-inflammatory drugs (NSAIDs) and (5) all subjects have to be the age of 18 or above. Those who were not fitting the criteria were excluded.

**Table 3:** Characteristics of *P*. *vivax*-exposed subjects from a malaria-endemic area in Thailand.

Characteristics	*P*. *vivax*-exposed subjects				Healthy subject
	Acutely infected subjects	Recovered subjects			
		3 months^a^	9 months^b^	12 months^c^	
Total (*n*)	27	21	15	14	32
Sex ratio (male:female)	1.6:1	2:1	0.8:1	1.3:1	0.5:1
Age (y)					
Mean (SD)	35.1 (12.6)	36.5 (14.2)	39.4 (13.2)	39.5 (13.1)	21.1 (0.82)
Range	18–63	18–63	22–63	22–63	20–24

^a^ 3 months: 2–3 months after recovery from *P*. *vivax* infection.

^b^ 9 months: 8–9 months after recovery from *P*. *vivax* infection.

^c^ 12 months: 12 months after recovery from *P*. *vivax* infection.

### Expression of recombinant PvRAMA protein

The 810 bp fragment of *pvrama* (PlasmoDB ID: PVX_087885) was amplified by PCR. The primers used for In-Fusion cloning were as follows: 5′-AAG GAG GCA GTG AAG GG-3′ and 5′-TTA ATT GGT GAA ACA TAA CAA TCC G-3′. The target gene was cloned into pEU plasmid DNA and the inserted nucleotide sequence confirmed using an ABI PRISM 310 Genetic Analyzer and a BigDye Terminator v. 1.1 Cycle Sequencing kit (Applied Biosystems, Foster City, CA, USA). The plasmid DNA was highly purified using a Maxi Plus Ultrapure plasmid extraction system (Viogene, Taipei, Taiwan). Briefly, purified plasmid DNA was eluted in 0.1× TE buffer (10 mM Tris–HCl, pH 8.0, 1 mM EDTA) and used for *in vitro* transcription and subsequent translation in a wheat-germ cell-free protein expression system (CellFree Sciences, Matsuyama, Japan) [45]. Recombinant PvRAMA protein was purified using a Ni-nitrilotriacetic acid agarose column (Qiagen, Valencia, CA, USA). Recombinant PvMSP1 protein was expressed described previously [46] and used for this study.

### ELISA measurement of cytokine production in PBMC cultures

Supernatants from PvRAMA-stimulated cultures were used to measured soluble cytokine protein using cytokine ELISA kits (BD OptEIA; BD Biosciences, San Diego, CA, USA). Anti-IL-2, IL-10, and anti-IFN-γ antibodies were immobilized onto plastic microwell plates according to the manufacturer’s protocol. Then, 50 μl of ELISA diluent was added to each well followed by 100 μl of culture supernatant. After 2 h incubation, the immobilized antibodies had specifically captured the soluble cytokine protein in the sample, and then the plates were washed to remove unbound materials. The captured cytokine proteins were detected with streptavidin-horseradish peroxidase conjugate mixed with biotinylated anti-human cytokine antibodies (detection antibodies). Thirty minutes following the addition of 100 μl of the chromogenic substrate tetramethylbenzidine (TMB) solution 50 μl of stop solution was applied. The level of colored product was measured with a spectrophotometer, as the OD at 450 nm. A standard curve or calibration curve was generated for each ELISA plate, which was used to calculate the cytokine concentration (typically pg of cytokine/ml).

### Flow cytometry

The phenotypes of cells responding to PvRAMA antigen were defined by staining for surface markers of T cells using monoclonal antibodies against CD3 (Alexa700), CD4 (PerCP Cy5.5) and CD8 (APC Cy7) plus monoclonal antibodies specific for IFN-γ (PE Cy7), IL-2 (APC), and IL-10 (PE), and analyzed by flow cytometry (BD FACS Canto II; Becton Dickinson, Oxford, UK). In brief, one million PBMCs were stimulated with 20 ng/ml PMA plus 1 μg/ml ionomycin (Sigma-Aldrich, St. Louis, MO, USA), or with 10 μg/ml recombinant PvRAMA antigen, or with medium alone. A protein transport inhibitor, 10 μl/ml brefeldin A, was also included in the cultures, which were kept at 37°C in 5% CO_2_ for 6 h. Cells were washed with washing buffer (PBS, 1% BSA, 0.1% NaN_3_) followed by staining with anti-CD3/CD4/CD8 for 15 min at 4°C, and then fixed with 0.5% paraformaldehyde solution at 4°C for 20 min. The cells were then permeabilized using the BD Cytofix/Cytoperm buffer system (BD Biosciences, San Jose, CA, USA) for 30 min at room temperature and labeled with anti-IL-2/anti-IFN-γ/anti-IL-10 for 20 min in the dark. After labeling, cells were washed and maintained in the buffer until data acquisition. Data were analyzed using FlowJo (v. 7.0; Tree Star Inc., San Carlos, CA, USA).

### Serological response against PvRAMA

To investigate the prevalence and stability of antibody responses to PvRAMA antigen, human plasma from 27 acutely infected *P*. *vivax* patients and follow-up samples taken at 3, 6, and 12 months after recovery were assayed by ELISA. Purified PvRAMA at 5 μg/ml or purified PvMSP1-19 antigen at 2 μg/ml was coated onto 96-well plates, and plates were covered and incubated at 4°C overnight. The plates were then washed three times by filling the wells with 200 μl of washing buffer (0.2% Tween-20 in PBS). The recombinant protein-coated wells were incubated with 100 μl of blocking buffer (5% nonfat dry milk in PBS) for 2 h at room temperature, and washed three times with washing buffer. One hundred microliters of plasma diluted 1:200 in blocking buffer was added to each well and incubated for 1 h at room temperature, then plates were rinsed at least five times with washing buffer. Goat anti-human IgG-alkaline phosphatase diluted 1:1000 in blocking buffer was added to each well, and incubated for 1 h. The plate was washed 7 times with washing buffer, and 2,2′-azino-bis(3-ethylbenzthiazoline-6-sulfonic acid) substrate (Sigma-Aldrich, St. Louis, MI, USA) was added. Absorbance was recorded at 405 nm at 1 h after addition of developer reagent. The OD values for duplicate wells per individual were averaged. A baseline OD was established using plasma from 32 samples from malaria-naïve Thai, and this control value was subtracted from the test OD values to standardize the assay. The cutoff value for seropositivity was an OD greater than the mean plus two SD of that for the naïve controls.

The prevalence of various IgG isotypes specific for PvRAMA or PvMSP1-19 antigen in sera from 27 acutely infected *P*. *vivax* patients and 16 negative serum samples were selected. Briefly, 5 μg/ml of PvRAMA or 2 μg/ml of PvMSP1-19 in PBS was added to 96-well ELISA plates and placed at 4°C overnight. The remaining protein-binding sites in the coated wells were blocked by adding 100 μl blocking buffer for 2 h at room temperature, and then the plates were incubated with 100 μl of each individual serum diluted 1:100 in blocking buffer. Horseradish peroxidase-conjugated anti-human IgG1, IgG2, IgG3, and IgG4 antibodies diluted 1:1000 in blocking buffer were used for detection. Production of a colored product developed after the addition of TMB enzyme substrate. The cutoff value was the mean plus two SD of the OD at 450 nm of all negative serum samples.

### Statistical analysis

The differences in individual cellular responses and stability of antibody levels in each individual were evaluated using the Wilcoxon matched-pairs signed-rank test for non-normal distributions. Comparison of antibody and IgG isotype levels and between unpaired groups (patients compared to recovery or patients compared to naïve controls) was performed using the Mann–Whitney *U* test. Spearman’s rank correlation test was used to evaluate the correlations between IgG isotypes. The prevalence of seropositive responders was compared using Fisher’s exact test (χ^2^). *P*-values <0.05 were considered significant. The statistical analysis was performed and graphs prepared using GraphPad Prism (v. 5; GraphPad Software, San Diego, CA, USA).

## Supporting Information

S1 Text Summary of raw data for figures and tables. (XLSX)

## Abbreviations

PvRAMA: *Plasmodium vivax* Rhoptry-associated membrane antigen
PBMCs: Peripheral blood mononuclear cells
ELISA: Enzyme-linked immunosorbent assay
PHA: Phytohemagglutinin
PMA: Phorbol myristate acetate
Th: helper T cells
