# Correction: Naturally-Acquired Immune Response against *Plasmodium vivax* Rhoptry-Associated Membrane Antigen

**DOI:** 10.1371/journal.pone.0153110

**Published:** 2016-03-31

**Authors:** Siriruk Changrob, Bo Wang, Jin-Hee Han, Seong-Kyun Lee, Myat Htut Nyunt, Chae Seung Lim, Takafumi Tsuboi, Patchanee Chootong, Eun-Taek Han

There is an error in the affiliation for the ninth author. Eun-Taek Hun is not affiliated with #3 but with #2 Department of Medical Environmental Biology and Tropical Medicine, School of Medicine, Kangwon National University, Chuncheon, Gangwon-do, Republic of Korea.
