# Correction: Correction: Naturally-Acquired Immune Response against *Plasmodium vivax* Rhoptry-Associated Membrane Antigen

**DOI:** 10.1371/journal.pone.0156011

**Published:** 2016-05-16

**Authors:** The PLOS ONE Staff

There is an error in the Correction published on March 31, 2016. The ninth author’s name is: Eun-Taek Han. The correct text is:

There is an error in the affiliation for the ninth author. Eun-Taek Han is not affiliated with #3 but with #2 Department of Medical Environmental Biology and Tropical Medicine, School of Medicine, Kangwon National University, Chuncheon, Gangwon-do, Republic of Korea.

The publisher apologizes for the error.
